# The efficacy and safety of fire needle therapy for COVID-19: Protocol for a systematic review and meta-analysis

**DOI:** 10.1097/MD.0000000000021873

**Published:** 2020-08-21

**Authors:** Min Liu, Hongqiu Zhu, Qian Xiong, Zhulan Zeng, Xinlei Xu, Min Ye, Yanling Zeng, Xiaodan Hu, Ying Zhu

**Affiliations:** a The Affiliated Hospital of Chengdu University of Traditional Chinese Medicine, Chengdu City, Sichuan Province, China; b Department of Gynecology, School of Medical and Life Sciences, Chengdu University of Traditional Chinese Medicine / Reproductive & Women-Children Hospital of Chengdu University of Traditional Chinese, Chengdu City, Sichuan Province, China.

**Keywords:** coronavirus disease 2019, COVID-19, fire needle, protocol, systematic

## Abstract

**Background:**

Coronavirus disease 2019 (COVID-19), a new type of coronavirus, first reported in Wuhan, China at the end of December 2019. As a result of the worldwide outbreak, the number of patients continues to increase. With multiple therapeutic interventions, more and more patients are recovering. Fire needle is used as an alternative therapy. At present, there are no relevant articles for systematic review and meta-analysis, so this study will evaluate the efficacy and safety of fire needle in the treatment of COVID-19 pneumonia.

**Methods:**

The following electronic bibliographic databases will be searched to identify relevant studies from December 2019 to December 2020: MEDLINE, PubMed, Embase, Cochrane library, Web of Science, China National Knowledge Infrastructure (CNKI), Chinese Technical Periodicals (VIP), Wan-fang data, Chinese Biological Medicine Database (CBM), and other databases. All included articles were randomized controlled trial without any language restrictions. Two reviewers will independently conduct cations retrieval, de-duplication, filtering, quality assessment, and data analysis by the Review Manager (V.5.3). Meta-analysis, subgroup analysis and/or descriptive analysis were performed on the included data.

**Discussion:**

This study will investigate the application of fire needle in the treatment or prevention of COVID-19, and provide a high-quality synthesis to evaluate whether fire needle is an effective and safe intervention for COVID-19.

**Systematic review registration:**

PROSPERO registration number: CRD 42020193703.

## 1 Introduction

A new type of coronavirus, first reported in Wuhan, China at the end of December 2019. Since then, it has been raging around the world. The rapid spread of the epidemic has attracted worldwide attention.^[1–4]^ On February 11, 2020, The World Health Organization (WHO) officially named novel Coronavirus Disease 2019 (COVID-19) as “COVID-19.”^[5]^ Since the outbreak of COVID-19, the number of infected and suspected cases around the world has risen rapidly and spread regionally. As of 21 July 2020, a total of 14,859,811 confirmed cases (currently 5,334,141 confirmed cases, 99% of them are in Mild Condition, 1% of them are Serious or Critical), 613,367 deaths and 8,912,303 cures are reported globally.^[6]^ Therefore, effective prevention and treatment are crucial in this situation.

In Diagnosis and Treatment Plan for COVID-19 (trial version 7) issued by the National Health Commission of China, it is noted that transmission through respiratory droplets and close contact are the primary means of transmission. Clinically, fever, fatigue and dry cough are the main manifestations.^[7]^ The Chinese Society of Acupuncture and Moxibustion has published the application of relevant protocols in the treatment of COVID-19, involving acupuncture, moxibustion, acupoint massage, and health maintenance techniques.^[8,9]^ Detailed guidelines have been proposed for suspected cases, mild cases, normal cases and convalescent patients.

Fire needle is a traditional Chinese medicine therapy that treats diseases by burning special needle until it turns red and quickly piercing the skin to the acupuncture point of the body.^[10]^ In the treatment of COVID-19, it holds the functions of clearing away heat and dampness, purging fire and detoxification, removing blood stasis, and harmonizing qi and blood.^[11]^ In combination with clinical stages and symptoms, it is mainly targeted at the health care population, mild and common cases as well as those in recovery stage. The acupoints of the du meridian, the lung meridian of the hand taiyin, the bladder meridian of foot Taiyang and the stomach meridian of Foot Yangming will be mainly used.^[8,9,12]^ The clinical indications of fire needle therapy are extensive, which can be applied to the general population. Three elements of “needle burning, acupoint accuracy, and nursing care” can be well mastered, which has a high application safety.

According to the published research, there is a lack of high-quality evidence on fire needle in the treatment of COVID-19. Therefore, this study is intended to evaluate the efficacy and safety of fire needle therapy for COVID-19.

## 2 Methods

### 2.1 Study registration

This systematic review protocol has been registered in the PROSPERO (CRD 42020193703) on 23 June 2020. The protocol refers to the guide book of Preferred Reporting Items for Systematic Reviews and Meta-Analyses Protocols (PRISMA-P). If there are any significant revisions, we will update the PROSPERO records.^[13]^

### 2.2 Search strategy

MEDLINE, PubMed, Embase, Cochrane library, Web of Science, China National Knowledge Infrastructure (CNKI), Chinese Technical Periodicals (VIP), Wan-fang data, Chinese Biological Medicine Database (CBM), and other databases will be searched. The search results will be limited to human studies only, and all included studies were randomized controlled trial (RCT) without any language restrictions. Furthermore, the reference lists of all identified studies will be examined to identify studies not captured by electronic searches. The key search terms are ([“novel coronavirus” OR “new coronavirus” OR “2019 novel coronavirus” OR “Coronavirus disease 2019” OR “2019 nCoV” OR “COVID-19”] AND [“fire needle”] AND [“randomized” OR “randomly” OR “clinical trial” OR “randomized controlled trial”]). These search terms are shown in Table 1. Combinations of Medical Subject Headings (MeSH) and text words will be used. The search strategy will follow the Preferred Reporting Items for Systematic reviews and Meta-analysis (PRISMA) guidelines.

**Table 1:**
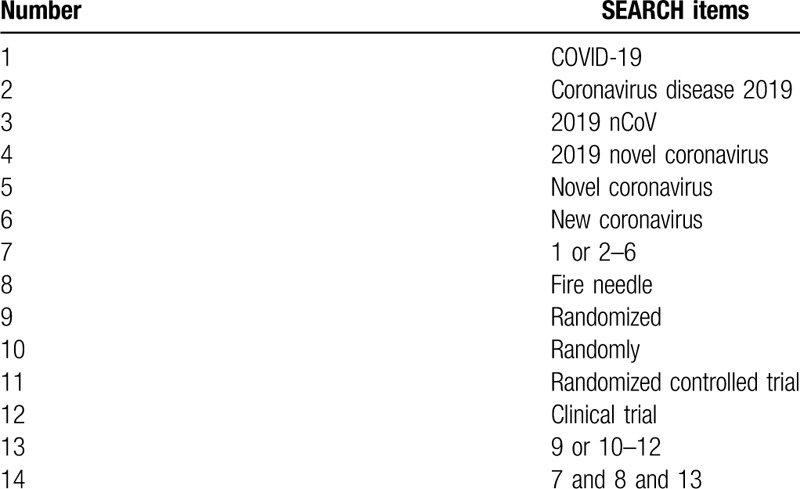
Search strategy for the PubMed database.

### 2.3 Inclusion criteria

#### 2.3.1 Type of study

We will include articles correlated to RCTs and credible clinical observation without randomization due to emergency. Due to language limitations, we searched Chinese and English articles to obtain more true and objective evaluation. All included articles were RCTs. If an experiment does not explain randomization, the article will be considered as high risk in random sequence generation.

#### 2.3.2 Types of participant

Patients diagnosed with COVID-19 and have no restrictions on disease stage, age, sex, ethnicity.

#### 2.3.3 Type of intervention

Fire needle alone or combined with one or more other pharmacological intervention will also be included. There will be no restrictions with respect to acupoint, frequency, duration, or follow-up time of treatment.

#### 2.3.4 Type of comparators

There will be no restrictions to the type of comparators. The comparators such as standard care, western medical therapies, Chinese medicine, etc.

#### 2.3.5 Types of outcomes

Primary outcomes were conversion rate from normal to severe, cure rate, mortality rate, Chest CT scans and nucleic acid detection of respiratory samples. Secondary outcomes were accompanying symptoms (such as myalgia, expectoration, runny nose, pharyngalgia, chest distress, dyspnea, crackles, headache, nausea, vomiting, anorexia, diarrhea) disappear rate, cellular inflammation level, average hospitalization time, adverse reactions, etc.

### 2.4 Exclusion criteria

1. life-threatening comorbidities that may lead to death during the follow-up period;
2. Duplicate data or data that cannot be extracted after contacting the corresponding author;
3. The full text cannot be obtained after contacting the corresponding author.

### 2.5 Data collection

#### 2.5.1 Data management

Citations will be managed using EndNote X9.1, and filtered independently by 2 authors. Statistical calculation will be performed by RevMan5.3 software and sensitivity analysis will be performed by Stata/SE 15.1 software.

#### 2.5.2 Data extraction

**Figure 1:**
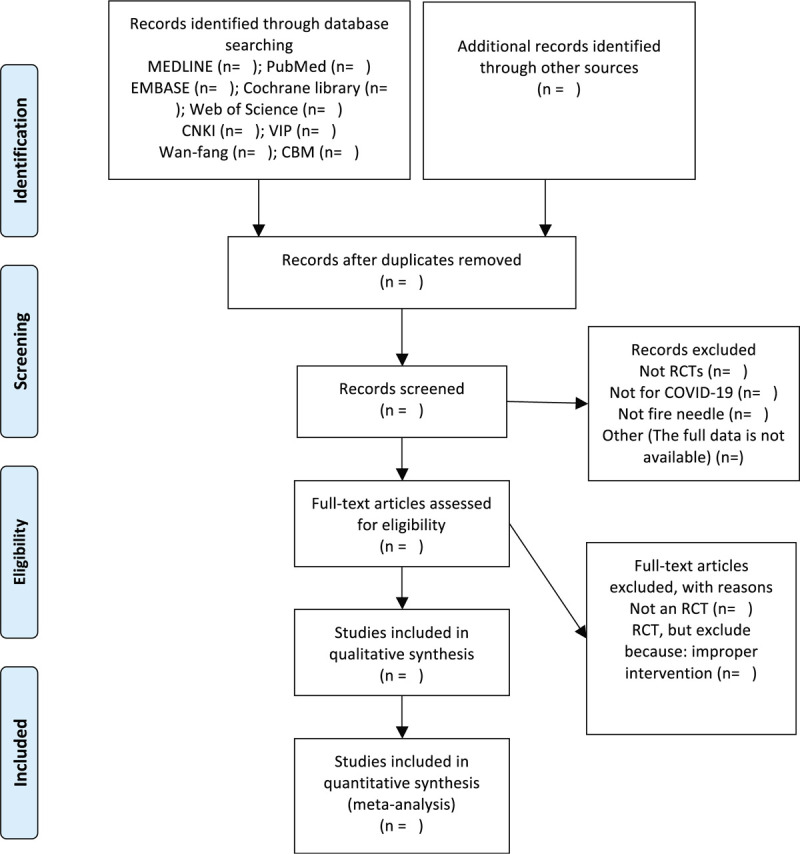
PRISMA flow diagram of the study process.

Studies meeting the eligibility criteria will be selected for inclusion. PRISMA flowchart was selected to show the study selection process (Fig. 1). The 2 authors will then extract relevant data using standardized methods, and will complete a data extraction table. Any differences of opinion between them will be resolved through discussion, or if this fails, a third author will arbitrate. The data extraction table will include the following information: the characteristics of the study (first author, title, publication year, language, and study design), participants (sample size, age, sex ratio, stage and severity of disease), intervention (duration, frequency, acupoint, follow-up time, types of control group), Outcome indicators (all outcomes, main conclusions), adverse reactions, and other information.

#### 2.5.3 Risk of bias assessment

The quality of the included studies will be evaluated independently by 2 authors using the Cochrane risk of bias tool. The risk of bias is divided into 3 levels (low risk, high risk, and unclear) based on the assessment in the following areas: sequence generation, allocation concealment, outcome evaluator and participant blindness, incomplete outcome data, selective outcome reporting, and other sources of bias. We will attempt to contact the relevant corresponding author for more detailed information to clarify unclear or inadequate items. Differences in opinion between the 2 authors over the risk of bias of a particular study will be resolved by discussion, with the participation of a third author, if necessary.

#### 2.5.4 Dealing with missing data

We will try our best to ensure the integrity of data. As data in the literature may be lost, we will contact the corresponding author by e-mail or other ways. If the missing data is not available, the study will be excluded from the study.

### 2.6 Statistical analysis

#### 2.6.1 Data synthesis

Data analysis and quantitative data synthesis will be performed using RevMan V.5.3. A meta-analysis will be conducted, if possible. For continuous data, if there is no heterogeneity, we will use mean difference or standard mean difference to measure the treatment effect of 95% confidence intervals. While dichotomous data will be represented as the relative risk (RR) with 95% CI. Heterogeneity between the studies will be assessed using the *Q* test and the *I*^2^ test. If the *I*^2^ value is less than 50%, the fixed effect model will be used for the data synthesis, whilst if *I*^2^ is between 50% and 70%, the random effects model will be used. If *I*^2^ values are higher than 75%, we will look for the possible causes of the heterogeneity from clinical and methodological perspectives, and will provide a descriptive analysis or subgroup analyses.

#### 2.6.2 Subgroup analysis

If heterogeneity is found, due to the above clinical trials, a subgroup analysis will be performed, with subgroups classified according to the outcomes of the data synthesis.

#### 2.6.3 Sensitivity analysis

If the studies included after subgroup analysis have significant heterogeneity, a sensitivity analysis will be conducted according to sample size, study design, heterogeneity quality, methodological quality and statistical model to exclude studies with quality defects and ensure the stability of analysis results.

#### 2.6.4 Assessment of reporting bias

In this analysis, if a sufficient number of studies are included, publication bias and other reporting bias will be assessed by creating funnel plots.

#### 2.6.5 Quality of evidence

Two independent reviewers will evaluate the quality of evidence for each outcome through the Grading of Recommendations Assessment, Development, and Evaluation (GRADE) system.^[14]^ Each outcome will be evaluated in five dimensions: limitations, inconsistencies, indirectness, inaccuracy, and publication bias. They will be rated as high, medium, low, or very low level.

#### 2.6.6 Ethics and dissemination

This study does not involve moral approval or ethical review, because we will use information from published studies. Our results will be published in a peer-reviewed journal based on PRISMA guidelines.

## 3 Discussion

In an epidemic of COVID-19, there is no effective drug. As a special Chinese medicine external therapy, fire needle therapy integrates acupuncture, direct moxibustion and acupuncture into one. It holds the characteristics of simplicity, convenience, speed and effect, and can be an effective supplement to Chinese medicine anti-epidemic treatment.^[15]^ If the symptoms of COVID-19 can be improved, it will bring benefits to COVID-19 patients. The purpose of this analysis is to gain an in-depth understanding of the efficacy and safety of fire needle in the adjuvant therapy of COVID-19, so as to provide reference for clinical treatment.

This is the first systematic review to evaluate empirical evidence for the use of fire needles for COVID-19 pneumonia. Summarize the use of fire needles in the treatment of COVID-19 patients, and assess the strength, safety, and limitations of the available evidence. In addition, it will achieve a level of quality as high as possible in reporting and methodology, guided by the PRISMA Statement ^[13]^ and A Measurement Tool to Assess systematic Reviews (AMSTAR) checklist.^[16]^ This scenario is based on the design and development of clinical trials currently registered, so it may have some limitations, but if these approaches change, we will update our proposed approach in our PROSPERO record. This review will serve to explore the potential role of the fire needle in treating or preventing viral infections that affect pneumonia.

## Author contributions

**Conceptualization:** Min Liu, Hongqiu Zhu.

**Data curation:** Qian Xiong.

**Investigation:** Min Liu, Hongqiu Zhu, Xinlei Xu, Ying Zhu.

**Methodology:** Min Liu, Hongqiu Zhu, Yanling Zeng.

**Project administration:** Min Liu, Hongqiu Zhu, Min Ye, Xiaodan Hu.

**Validation:** Zhulan Zeng.

**Writing – original draft:** Min Liu.

**Writing – review & editing:** Min Liu, Hongqiu Zhu.

## References

[1] Bulut C Kato Y Epidemiology of COVID-19. Turk J Med Sci 2020;50 (SI-1):563–70. 32299206 10.3906/sag-2004-172 PMC7195982

[2] Huang C Wang Y Li X Clinical features of patients infected with 2019 novel coronavirus in Wuhan, China. Lancet 2020;395:497–506. 31986264 10.1016/S0140-6736(20)30183-5 PMC7159299

[3] Li J Gong X Wang Z Clinical features of familial clustering in patients infected with 2019 novel coronavirus in Wuhan, China. Virus Res 2020;286:198043. 32502551 10.1016/j.virusres.2020.198043 PMC7265838

[4] Munster VJ Koopmans M van Doremalen N A novel coronavirus emerging in China - key questions for impact assessment. N Engl J Med 2020;382:692–4. 31978293 10.1056/NEJMp2000929

[5] WHO. WHO Director-General's remarks at the media briefing on 2019-nCoV on 11 February 2020. February, 11 2020. https://www.who.int/dg/speeches/detail/who-director-general-s-remarks-at-the-media-briefing-on-2019-ncov-on-11-february-2020 [accessed July 21, 2020].

[6] Worldometer. COVID-19 coronavirus pandemic. July 21, 2020. https://www.worldometers.info/coronavirus/ [accessed July 21, 2020].

[7] Chinese Health Commission Diagnosis and treatment for COVID-19 (trial version, 7). Chin Med 2020;15:801–5.

[8] Liu W Guo S Wang F Interpretation of guidance on COVID-19 acupuncture intervention by the Chinese acupuncture society (second edition). World J Acupunc Moxib 2020;30:1–4. 10.1016/j.wjam.2020.03.005 PMC7118592 32292259

[9] Shi X Tong X Sun G Guidelines for COVID-19 acupuncture intervention (second edition). Chin Acupunct Moxib 2020;40:462–3.

[10] Li Y Li H Jin Z Reverse therapy with fire needle treats heat syndrome. Chin Acupunct Moxib 2019;39:296–8.

[11] Cao Y Wang H Wen H Discussion on the application of fire needle intreating heat diseases. China's Naturop 2020;28:21–3.

[12] Lin Y Zhang Y Wei Y Exploration and thinking of fire needle therapy for prevention and treatment of COVID-19. Chin Acupunct Moxib 2020;40:693–6.

[13] Shamseer L Moher D Clarke M Preferred reporting items for systematic review and meta-analysis protocols (PRISMA-P) 2015: elaboration and explanation. BMJ 2015;350:g7647. 25555855 10.1136/bmj.g7647

[14] Balshem H Helfand M Schunemann HJ GRADE guidelines: 3. Rating the quality of evidence. J Clin Epidemiol 2011;64:401–6. 21208779 10.1016/j.jclinepi.2010.07.015

[15] Liu B Wang H Zhou Z Analysis on the theory and clinical ideas of acupuncture for the prevention and treatment of coronavirus disease 2019. Chin Acupunct Moxib 2020;40:571–5. 10.13703/j.0255-2930.20200305-k0004 32538003

[16] Shea BJ Reeves BC Wells G AMSTAR 2: a critical appraisal tool for systematic reviews that include randomised or non-randomised studies of healthcare interventions, or both. BMJ 2017;358:j4008. 28935701 10.1136/bmj.j4008 PMC5833365

